# AFF4 Predicts the Prognosis of Colorectal Cancer Patients and Suppresses Colorectal Cancer Metastasis *via* Promoting CDH1 Expression

**DOI:** 10.3389/fonc.2022.797392

**Published:** 2022-02-09

**Authors:** Yi Fang, Hua Cao, Xiaoyong Gong, Yanqing Chen, Yugang Zhuang, Shuqin Zhou, Yuanzhuo Chen, Yimei Jiang, Xiaopin Ji, Hu Peng, Xiaoqian Jing

**Affiliations:** ^1^ Emergency Department, Shanghai Tenth People’s Hospital, Shanghai, China; ^2^ Department of General Surgery, Ruijin Hospital, Shanghai Jiao Tong University School of Medicine, Shanghai, China

**Keywords:** AF4/FMR2 family member 4 (AFF4), colorectal cancer (CRC), super elongation complex (SEC), epithelial-mesenchymal transition (EMT), CDH1

## Abstract

**Introduction:**

AF4/FMR2 family member 4 (AFF4) is a core component of super elongation complex (SEC) and regulates the transcription elongation of many genes. AFF4 depletion or amplification is associated with multiple cancers, but its role in colorectal cancer (CRC) has not been investigated so far.

**Methods:**

qRT-PCR and Western blot analyzed AFF4 expression in the paired clinical CRC tissues. The patients’ overall survival curve was determined using the Kaplan-Meier plotter. *In vitro* experiments, such as cell proliferation, migration, and invasion, were used to preliminarily ascertain the role of AFF4 in CRC. A CRC cell liver metastasis animal model was well established. Livers were harvested and examined histologically by a series of indicators, such as tumor nodules, liver weight, ALT/AST activity, and tumor cell identification by hematoxylin-eosin (HE) staining.

**Results:**

We firstly examined the expression of AFF4 in colorectal cancer and normal tissues by collecting paired CRC tissues and adjacent normal tissues, revealing that AFF4 was significantly downregulated in CRC patients and lower expression of AFF4 was correlated with poor prognosis. Next, we observed that presence or absence of AFF4 in CRC cells had no effect on cancer cell proliferation, while AFF4 depletion significantly promoted the migration or invasion of CRC cells *in vitro*. Furthermore, we confirmed that AFF4 deficiency enhanced the metastatic capacity of CRC cells *in vivo*. Mechanistically, we found that AFF4 upregulated the transcription of *CDH1* gene, which encodes E-cadherin and suppresses the epithelial-mesenchymal transition (EMT). Knockdown of AFF4 interfered with *CDH1* transcription, resulting in downregulation of E-cadherin expression and the progression of CRC. Moreover, restored *CDH1* expression could rescue the phenotype of CRC cells without AFF4.

**Conclusions:**

Collectively, our data demonstrated that AFF4 served as a significant novel regulator of CRC *via CDH1* transcriptional regulation and a potential effective therapy target for patients with CRC.

## Introduction

As the global population ages, the incidence of colorectal cancer is forecasted to continue to increase each year, with the number of new cancer cases expected to reach nearly 30 million worldwide by 2040, increasing the cancer burden by 50%. According to the latest global cancer data for 2020 released by the International Agency for Research on Cancer (IARC), there were more than 1.9 million new colorectal cancer (CRC) cases and 935,000 deaths worldwide, accounting for about one-tenth of cancer cases and deaths. CRC is the third most common cancer, but it has the second highest death rate (1, 2). China leads the world in both new cancer cases and deaths, accounting for 30% of all cancer deaths worldwide. In the first-tier cities of China, colorectal cancer is the most rapidly increasing incidence of malignant tumor in recent years, second only to lung cancer, ranking the second malignant tumor, with up to 500,000 new cases every year, and still increasing at an annual rate of 4%–5%. Although CRC usually takes 10–20 years from the incubation period to the clinical stage of cancer, due to the insidious onset of CRC, more than 90% of cancers may have no obvious symptoms during the incubation period, and many CRC patients are already advanced or accompanied by metastasis at the time of diagnosis, thus missing the best treatment opportunity (3). At present, the treatment mainly depends on surgical excision combined with chemotherapy and radiotherapy and targeted therapy, but for patients with advanced CRC, both drug resistance rate and recurrence rate are high, and survival rate is difficult to improve. Therefore, it is of great scientific and social significance to carry out basic research on CRC, discover the molecular mechanism affecting distant metastasis of colorectal cancer, explore potential intervention methods, and screen new targeted drugs, so as to provide scientific basis for early effective prevention and precision medicine of colorectal cancer and realize personalized treatment (4–6).

Recently, transcription factor elongation regulation, especially super elongation complex (SEC), has been characterized as an important regulator of cancer progression. Transcriptional elongation is a tightly regulated step during gene expression. The SEC family regulates gene expression through rapid transcriptional induction and plays an important role in gene activation in normal physiology and in cancer development and pathogenesis (7, 8). The SEC is required for the expression of MYC in both two hematology AML and ALL. The translocation of any of the SEC components to the amino-terminal domain of mixed-lineage leukemia (MLL) abnormally stabilizes the localization of the SEC at MLL complex-targeted genes, including HOXA9 and HOXA10, leading to aggressive acute leukemia. Therefore, the SEC could be a potential target for the treatment of cancers with MYC overexpression (9). Otherwise, there was structure basis of SEC to support the mechanism of prompting gene expression. Viral trans-activator of transcription (Tat), for example, recruits SEC into the HIV-1 long terminal repeat sequence (LTR) to activate the provirus expression in host cells, leading to an active HIV-1 infection process (10).

The SEC contains the eleven–nineteen lysine-rich leukemia gene (ELL) family members ELL1 and ELL2, the MLL translocation partners AF4/FMR2 family member AFF1 (also known as AF4), AF4/FMR2 family member 4 (AFF4), ALL1-fused gene from chromosome 9 (AF9), ENL, and P-TEFb (the Pol II elongation factor) (11–13). AFF4, which directly interacts with P-TeFb and AF9 or ENL, becomes the scaffold for assembling SEC and is a core component necessary for SEC stability and activity. AFF4 contains conserved N- and C-terminal domains, an ALF homology region, and a serine-rich transactivation domain that was involved in transcriptional activation. As an essential component of SEC, AFF4 can bind to DNA directly and regulate the transcription elongation of many genes (11, 14–16). Depletion of AFF4 in leukemia cells results in reduced expression of MLL chimeric target genes. In addition, AFF4 promotes tumorigenesis and tumor-initiation capacity of head and neck squamous cell carcinoma and bladder cancer (17, 18). Although there are numerous connections linking diseases with AFF4, however, whether colorectal cancer progression is associated with AFF4 expression remains unknown.

This research aimed to investigate the role of AFF4 on colorectal cancer by measuring proliferation, migration, as well as invasion capacity *in vitro* and *in vivo*. Our study analyzed AFF4 expression in CRC tissues to explore its role in CRC progression, revealing that AFF4 was downregulated in CRC tissues and reversely predicted poor prognosis. Therefore, we hypothesized that AFF4 played an inhibitory role in the progression of CRC and may provide a new target for the treatment of CRC.

## Materials and Methods

### Database

We mined the expression of AFF4 in colorectal cancer tissues and adjacent tissues from the Gene Expression Profiling Interactive Analysis (GEPIA) database. The Cancer Genome Atlas Program (TCGA) colorectal cancer patients’ survival data for AFF4 were downloaded from www.tcgaportal.org and www.proteinatlas.org. Patients were then divided into two groups, low and high, according to the expression level using the best cutoff. Kaplan–Meier survival curves were reconstructed using R. The log-rank *p*-value was reported.

### Patients and Tissue Specimens

Colorectal cancer tissues and their matched adjacent tissues were collected from colorectal cancer patients who were pathologically diagnosed from Ruijin Hospital, Shanghai Jiao Tong University School of Medicine, from September 2018 to September 2020. All patients were not treated with chemotherapy, radiotherapy, and immunotherapy before operation. After receiving the tissue samples, RNA storage solution was put into the patient immediately, 4°C overnight, then transferred the specimen to freeze and stored at an −80°C refrigerator. All patients and their families who were included in the experiment signed the informed consent and agreed to participate in the experiment. The research content and implementation plan of this experiment strictly comply with the declaration of Helsinki and have been approved by the ethics committee of Ruijin Hospital Affiliated to Shanghai Jiaotong University School of Medicine and the ethics committee of the 10th People’s Hospital Affiliated to Tongji University of Shanghai.

### RNA extraction and Quantitative RT-PCR

Total RNA was extracted with an RNA high-purity total RNA rapid extraction kit (Qiagen, Hilden, Germany). cDNA was prepared using a GoScript reverse transcription system (Promega, Madison, WI, USA). Quantitative RT-PCR (qRT-PCR) was performed using SYBR PCR premixture (Promega) under the following conditions: 5 min at 95°C followed by 38 cycles at 95°C for 30 s, 60°C for 40 s, and 72°C for 1 min using an ABI 7500 fast system. Data were normalized to the expression of the control gene (β-actin) for each experiment. Data represent the mean ± SD of three independent experiments. The sequences of primer pairs used for qRT-PCR are listed in **Supplementary Table 1**.

### Construction of AFF4 Overexpression and Knockdown Cells

The plasmid and lentivirus particles for AFF4 overexpression or knockdown were designed by Shanghai GenePharma Co., Ltd. (Shanghai, China). The pGIPZ control or pGIPZ AFF4 shRNA was generated by inserting a small hairpin RNA targeting GFP or human AFF4 ORF (NM_014423.4) with a sequence 5′-cccaagcctacagtaccacca-3′ for #1 or 5′-cctccatctgcaccaactctt-3′ for #2 into the pGIPZ vector. HCT116 and LoVo cells were transduced with Lenti-GFP (EV) or Lenti-AFF4 OV (AFF4 OV) at 20 MOI in the presence of 8 μg/ml of polybrene (Sigma, St. Louis, MO, USA). HT29 and DLD-1 cells were infected with Lenti-control (shNT) or Lenti-shAFF4#1 (shAFF4#1) and Lenti-shAFF4#2 (shAFF4#2) at 20 MOI in the presence of 8 μg/ml polybrene. Cell selection was continuously performed in puromycin for 36 h and passages were performed (2 μg/ml).

### Cell Proliferation Assay

CCK8 was used to determine the proliferation ability of cells: cells in logarithmic growth phase were inoculated into 96-well plates (8 × 10^3^/200 μl) Each group had 7 multiple holes. The operation was carried out according to the instructions of CCK-8: 7 multiple wells were set in each well, the original medium was discarded at 0, 24, 48, 72, and 96 h, and the medium containing 10% (volume fraction) CCK-8 reaction solution was added again 50 μl. The absorbance of each well was detected at 450 nm by ELISA.

### Transwell Migration Assay

Measurement of cell migration ability: the cells were digested and centrifuged and counted under the microscope, and 1 × 10^4^ cell suspensions per well were seeded in the upper chamber of Transwell chamber. Transwell chamber was placed in 24-well plate, and 500 cells were added in the upper chamber with 100 μl DMEM medium. After 24 h of continuous culture in the incubator, the chamber was taken out and placed in 4% paraformaldehyde solution; the cells were fixed for 30 min and stained with crystal violet at room temperature for 10 min. The upper layer of cells that did not pass through the ependyma were gently wiped off with cotton swabs and washed twice with PBS. Under 200 times microscope, five high-power fields were randomly selected to count the number of cells passing through the membrane, and the average number was taken as the number of cells passing through the chamber in each group, so as to evaluate the migration ability of cells.

### Transwell Invasion Assay

The invasion ability of cells was measured: the experimental steps were basically the same as the migration experiment. Only 2 h before the experiment, Matrigel was diluted to a concentration of 1:8 (volume ratio) and placed in the upper part of the chamber and placed in 37°C. The results showed that the CO_2_ content was 5% for 2 h, and the subsequent experiments were carried out after the matrix glue solidified.

### Experimental Liver Metastasis Model

Athymic BALB/c 4–6-week-old nude mice were purchased from Shanghai SLRC Co., Ltd. (Shanghai, China) and treated under specific pathogen-free conditions. Animals were allowed a 1-week adaptation period after arrival. All experiments were performed according to the institutional ethical guidelines on animal care and approved by Ruijin Hospital, Shanghai Jiao Tong University School of Medicine. Mice were injected with HT29-shNT or HT29-shAFF4#1 cells with 2 × 10^6^/100 µl/mouse and five mice per group *via* tail vein. After 2 months, mice were sacrificed and livers were sorted. Visible liver metastatic nodules were counted or detected in paraffin-embedded sections stained with H&E. Liver weight was also measured.

### Xenograft Study

In brief, 2 × 10^6^ HT29 shNT or shAFF4 cells were subcutaneously injected into the left dorsal flank of randomized 15-week-old female athymic nude mice. After inoculation for 30 days, mice were euthanized, and tumors were dissected. Differences in weight, proliferation, and apoptotic rate of xenograft tumors or the cells were statistically analyzed.

### Statistical Analysis

SPSS 13.0 software was used to analyze the experimental data. Each experiment was repeated three times, and the results were calculated by means ± standard deviation. Univariate analysis of variance was used for comparison between groups, and LSD *t*-test was used for pairwise comparison within groups. Paired *t*-test and Chi-square test were used to compare cancer and adjacent tissues. *p* < 0.05 was statistically significant.

## Results

### AFF4 Is Downregulated in CRC Tissue and Reversely Predicts Poor Prognosis

**Figure 1:**
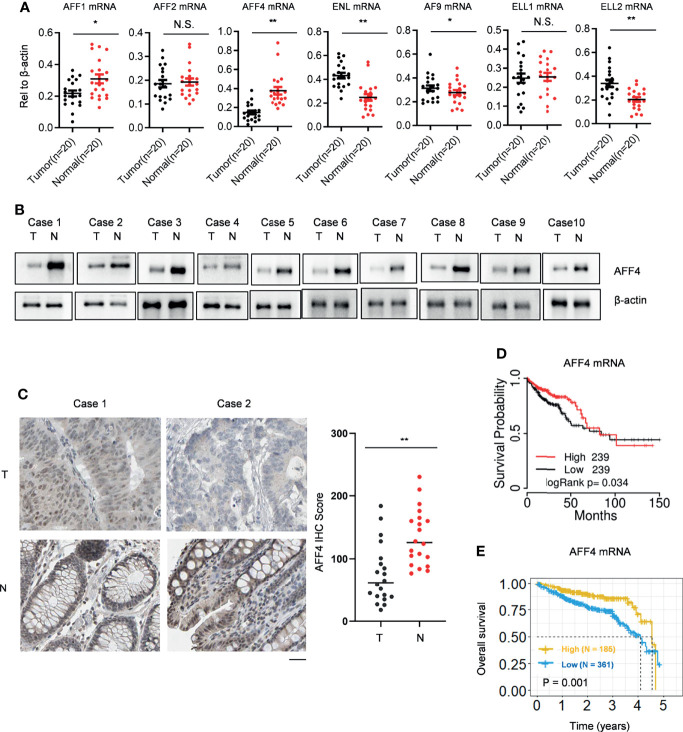
AFF4 is downregulated in CRC tissue and reversely predicts poor prognosis. **(A)** SEC components, including AFF1/2/4, ENL/AF9, and ELL1/2 are listed, and their gene expressions were determined by qRT-PCR in clinical colorectal cancer and adjacent tissues collected by Ruijin Hospital. Total RNA of 20 pairs of colorectal cancers and their adjacent normal tissues were extracted, and β-actin was performed as an internal control. Primers against mRNA level of above genes are listed in *Materials and Methods* section. *p < 0.05; **p < 0.01; NS, not significant. **(B)** Total protein in 20 pairs of colorectal cancers and their adjacent normal tissues were extracted by strong lysis buffer and centrifugation, and β-actin was performed as an internal control. Indicated antibodies testing AFF4 protein level is listed in *Materials and Methods* section. **(C)** Immunohistochemistry (IHC) was performed to detect AFF4 distribution in the 20 pairs of colorectal cancers and their adjacent normal tissues using antibody specifically against AFF4 in tissue level (left). The IHC score was calculated and analyzed in right panel. The results of IHC assay and the statistic method of IHC are described in the *Materials and Methods* section. **(D)** The survival time information of 478 CRC patients were extracted from TCGA database and a median cutoff separately divided these patients into two groups: high (*N* = 239) and low (*N* = 239). The longest statistic time is 150 months. **(E)** The survival time information of 546 CRC patients in 5 years were extracted from the Human Protein Atlas database and a best cutoff separately divided these patients into two groups: high (*N* = 185) and low (*N* = 361). The value of best cutoff is 7.33.

SEC comprised several cellular proteins including the scaffold proteins AFF1, AFF2, AFF4, ENL, and AF9, the elongation factors ELL1 and ELL2, and the positive transcription elongation complex P-TEFb. In order to explore the role of SEC in colorectal cancer, we first collected 20 paired CRC tissues and adjacent normal tissues. Using qRT-PCR, we observed that in CRC tissues, AFF4/1 were downregulated while ENL/AF9/ELL2 were upregulated (**Figure 1A**). SEC is a potent activator of transcriptional elongation and was regarded as an oncogenic role according to previous studies (11, 17, 19); we are more interested in the probable suppressive role of SEC components in CRC. Hence, we focused on AFF4 as it dramatically decreased in mRNA level in CRC tissues, which was ascertained by Western blot (**Figure 1B**). Furthermore, we also tested AFF4 expression and distribution using immunohistochemistry (IHC) and found AFF4 was actually downregulated in CRC tissues and located in the nucleus (**Figure 1C**). Except our home collected patients’ samples, we again searched GEPIA database, which includes RNA sequencing expression data of 9736 tumor samples and 8587 normal samples from TCGA and GTEX, to compare the expression of AFF4 in CRC tissues and normal tissues. The results showed that AFF4 was significantly downregulated in colorectal cancer tissues (**Supplemental Figure 1A**).

To explore the relationship between the expression of AFF4 and the prognosis of colorectal cancer patients, we collected RNA sequencing data from TCGA database and the Human Protein Atlas database, respectively (**Supplemental Figures 1B, C**), and analyzed the correlation of low expression of AFF4 and the survival time of colorectal cancer patients by Kaplan-Meier. The results showed that the prognosis of patients with low level of AFF4 was poor, while the survival time of patients with high expression of AFF4 was relatively long. It is worth noting that especially in the first 3 years, patients with high expression of AFF4 had a survival rate of more than 85%, while patients with low expression of AFF4 showed a survival rate of less than 75% (**Figures 1D, E** **)**. The results suggested that AFF4 may play an important role in colorectal cancer suppression.

### AFF4 Regulates CRC Cell Migration and Invasion But Not Proliferation

**Figure 2:**
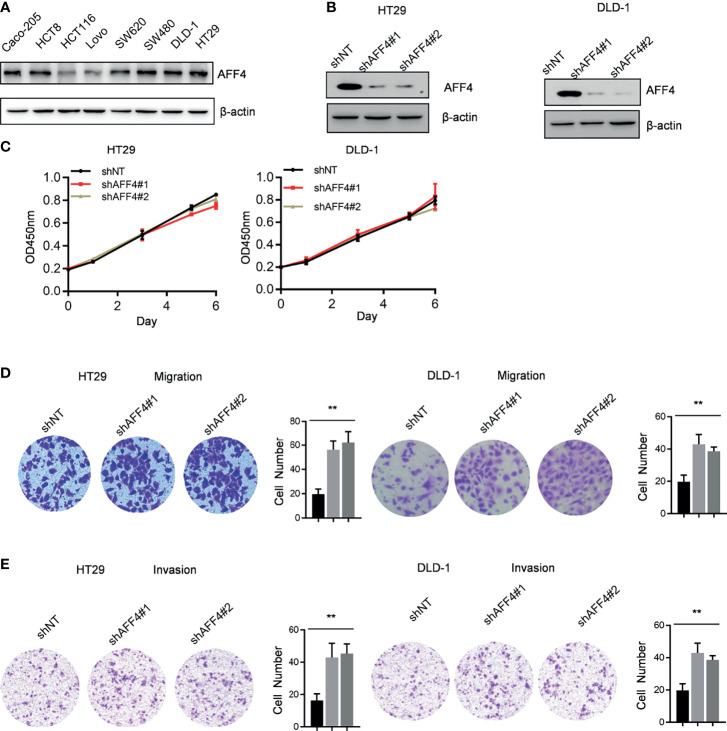
AFF4 depletion promotes CRC cell migration and invasion but not proliferation. Data are from three independent experiments. Data are presented as the mean ± SD or SEM and are analyzed by unpaired *t*-tests or two-way ANOVA. ^**^ *p* < 0.01. **(A)** Western blot assay was performed to determine AFF4 protein level in eight colorectal cancer cell lines. β-Actin was performed as an internal control. **(B)** Western blot assay was performed to determine AFF4 knockdown efficiency in HT29 and DLD-1. β-Actin was performed as an internal control. Sh, small hairpin; NT, nontarget. **(C)** CCK-8 assay was performed to measure the effect of AFF4 knockdown on HT29 and DLD-1 cell proliferation. A total of 20,000 cells were seeded in 48-well plates at the start point and collected at the indicated time points. The calculation value of cell number was readout by OD450 nm using Microplate System. **(D, E)** Cell migration or invasion was performed by seeding 50,000 HT29 and DLD-1 cells expressing shNT, shAFF4#1, or shAFF4#2 in the serum-free medium, and migrated **(D)** or invasive **(E)** cells through the 8-μm hole were fixed by 80% cold methanol and stained by crystal violet. Of note, the invasion assay was performed by adding Matrigel in the 8-μm hole. The calculation of cell number was executed by differential interference contrast microscope (DIC).

A panel of human CRC cell lines expressing different levels of AFF4 was obtained from the American Type Culture Collection (ATCC), and a representative blot of AFF4 expression level in each cell line is shown in **Figure 2A**. To examine the effects of AFF4 depletion on tumorigenesis-related processes in HT29 and DLD-1 cells which expressed higher level of AFF4, we knocked down AFF4 in HT29 and DLD-1 cells using two unrelated small hairpin RNAs, and the knockdown efficiency was verified by Western blot (**Figure 2B**). However, AFF4 depletion did not accelerate or inhibit the proliferation of HT29 and DLD-1 cells, measured by CCK-8 for 6 days (**Figure 2C**). We further tested the migration and invasion ability of HT29 and DLD-1 cells after AFF4 depletion. Interesting, the absence of AFF4 significantly promoted the migration and invasion of HT29 and DLD-1 cells after 24 h, suggesting that AFF4 may have the function of suppressing colorectal cancer metastasis (**Figures 2D, E** **)**.

**Figure 3:**
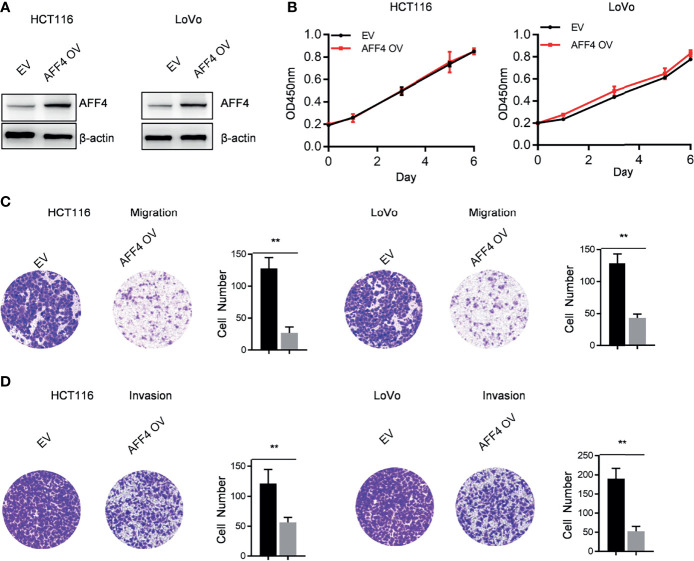
Overexpression of AFF4 hampers CRC cell migration and invasion but not proliferation. Data are from three independent experiments. Data are presented as the mean ± SD or SEM and are analyzed by unpaired *t*-tests or two-way ANOVA. ^**^ *p* < 0.01. **(A)** Western blot assay was performed to determine overexpression efficiency in HCT116 and LoVo cells. β-Actin was performed as an internal control. EV, empty vector, as a blank control; OV, overexpression. **(B)** CCK-8 assay was performed to measure the effect of AFF4 overexpression on HT29 and DLD-1 cell proliferation. A total of 20,000 cells were seeded in the 48-well plate at the start point and collected at the indicated time points. The calculation value of cell number was readout by OD450 nm using Microplate System. **(C, D)** Cell migration or invasion was performed by seeding 50,000 HT29 and DLD-1 cells expressing empty vector or AFF4 in the serum-free medium, and migrated **(C)** or invasive **(D)** cells through the 8-μm hole were fixed by 80% cold methanol and stained by crystal violet. Of note, the invasion assay was performed by adding Matrigel in the 8-μm hole. The calculation of cell number was executed by differential interferencecontrast microscope (DIC).

In addition to AFF4 depletion, we overexpressed AFF4 in HCT116 and LoVo cells which expressed lower level of AFF4 (**Figures 2A** and **3A**), and the role of AFF4 overexpression on CRC cell proliferation, migration, and invasion was consequentially analyzed. Similarly, the results showed that AFF4 overexpression has no effect on the proliferation of colorectal cancer cells (**Figure 3B**), but significantly inhibited the migration and invasion of HCT116 and LoVo cells (**Figures 3C, D** **)**. Hence, *in vitro* experiments such as migration and invasion assay robustly proved AFF4 displayed a suppressive role in cell mobility.

### AFF4 Depletion Has No Effect on Solid Tumor Formation

Using *in vitro* experiments, we observed AFF4 depletion or overexpression showed no effect on CRC cell proliferation. We wondered whether AFF4 contributes to tumor growth or formation *in vivo*, as tumor growth supported tumor progression, such as metastasis. To examine whether loss of AFF4 exerts effects on the formation of solid tumors, we transplanted HT29 cells with genetic manipulation of AFF4 depletion and rescued simultaneous expression of both HPRT1 and IMPDH2 into the left groin of nude mice. After 30 days, we excised tumors from nude mice and imaged solid tumors. As shown in **Supplemental Figure 2A**, loss of AFF4 has not impaired tumor formation, in detail, there was no difference in solid tumor volume or weight (**Supplemental Figure 2A**; **Figure 2B**). Furthermore, IHC was performed using an antibody against Ki67, which is indicative of cell proliferation, in solid tumors. We observed that AFF4 depletion did not exert an effect on cell proliferation (**Supplemental Figure 2C**). Also, TUNEL staining showed us AFF4 depletion did not exert an effect on cell apoptosis (**Supplemental Figure 2D**).

### AFF4 Depletion Conferred CRC Cells a More Aggressive Capacity of Metastasis *In Vivo*

**Figure 4:**
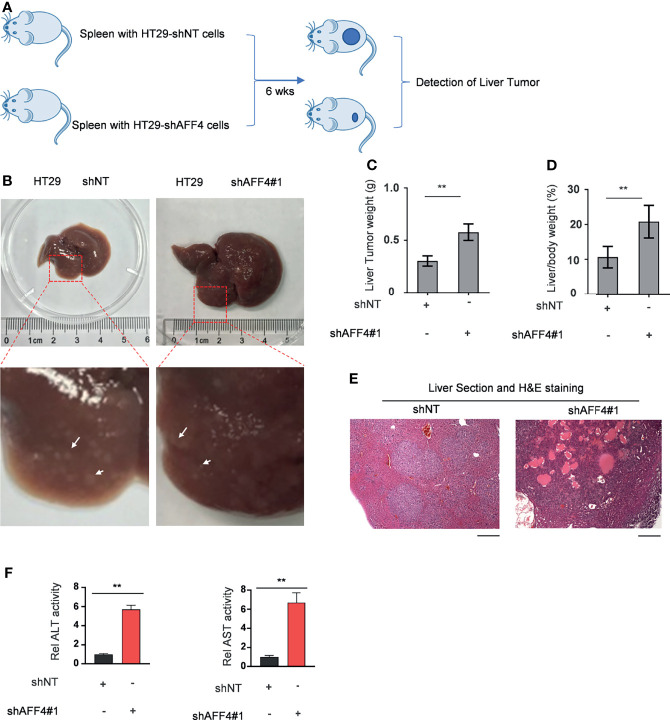
AFF4 depletion conferred CRC cells more aggressive capacity of metastasis to liver. Data are presented as the mean ± SD or SEM and are analyzed by unpaired *t*-tests. ^**^ *p* < 0.01. **(A)** The work follows a liver metastasis model. A total of 50,000 HCT116 cells expressing shNT or shAFF4 were injected into the spleen of nude mice (*N* = 4), then after 6 weeks, the metastatic lesions in the liver of nude mice were observed and tumor samples were separately collected. **(B)** After the mice were sacrificed, the representative images of liver metastases nodules were photographed and enlarged, then marked with a white arrow. **(C–F)** The analysis of liver metastasis with AFF4 genetically manipulated HCT116 cells. The weight of liver with metastases nodules was first measured and calculated **(C)**. The weight ratio of liver to the whole body of mice after metastasis was then calculated **(D)**. Hematoxylin-eosin (H&E) staining was performed to show the tumor cells in the liver metastasis nodule specimens **(E)**. The enzymatic activities of glutamic-pyruvic transaminase (ALT) and glutamic oxalacetic transaminase (AST) were measured using a kit to indicate the liver function. The value of enzymatic activities in the shAFF4 group was normalized to the control group **(F)**. Scale bar, 1 mm.

In addition, we evaluated the contribution of AFF4 depletion in CRC metastasis *in vivo* using hepatic metastasis mouse model (**Figure 4A**). After 6 weeks for spleen injection with 50,000 cells, as shown in **Figure 4B**, the control tumor cells HT29 developed a tiny hepatic metastasis, while the HT29 cells with AFF4 depletion had a vast hepatic metastasis (**Figure 4B**). Meanwhile, the weight of liver was also examined to test whether the metastatic tumor cells directed the swollen part. Results display that depletion of AFF4 in HT29 cells induced an increased liver metastasis and then generated a more serious liver swell (**Figures 4B–D**). Furthermore, the metastatic tumors were validated by hematoxylin-eosin (HE) staining of dissected hepatic nodules (**Figure 4E**). Finally, we tested the enzymatic activities of glutamic-pyruvic transaminase (ALT) and glutamic oxalacetic transaminase (AST), which indicate the liver function. As shown in **Figure 4E**, comparing with the control group, the enzymatic activities of ALT and AST in the group of metastatic mice liver increased about 6–8 times (**Figure 4F**). Taken together, our *in vivo* results suggested AFF4 depletion conferred CRC cells a more aggressive capacity of metastasis.

### AFF4 Promotes CDH1 Expression Which Impairs the Cell Migration and Invasion *In Vitro*

**Figure 5:**
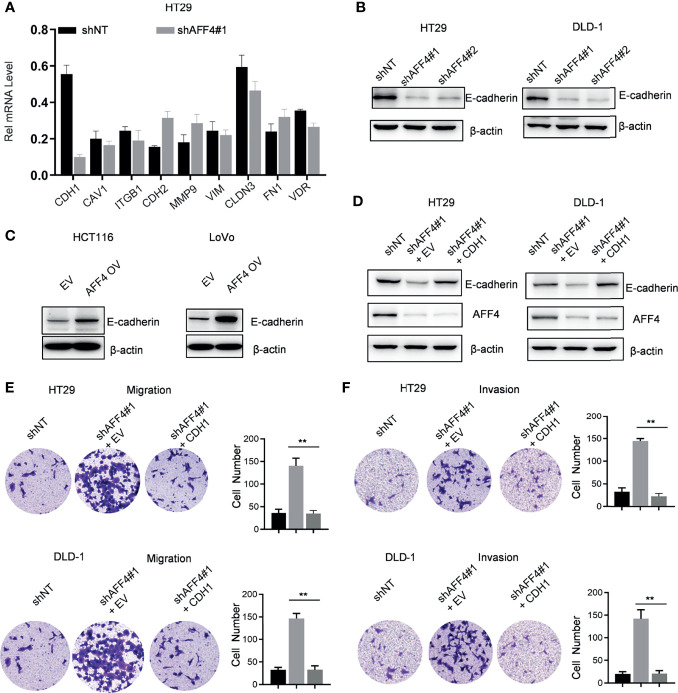
AFF4 promotes CDH1 expression which impairs the cell migration and invasion *in vitro*. Data are from three independent experiments. Data are presented as the mean ± SD or SEM and are analyzed by unpaired *t*-tests or two-way ANOVA. ^**^ *p* < 0.01. **(A)** A total of 9 genes related to cell migration or invasion were listed, and their gene expression were determined by qRT-PCR in the cell expression shNT or shAFF4#1. β-Actin was performed as an internal control. **(B)** Western blot assay was performed to test CDH1 expression in the HT29 or DLD-1 cells with or without AFF4. Sh, small hairpin; NT, nontarget. **(C)** Western blot assay was performed to test CDH1 expression in the HCT116 or LoVo cells with or without overexpression of AFF4. EV, empty vector, as a blank control; OV, overexpression. **(D)** Western blot was performed to test the successfully restored expression of CDH1 in the cells HT29 or DLD-1 cells with or without AFF4. **(E, F)** Cell migration or invasion was performed by seeding 50,000 HT29 and DLD-1 cells expressing shNT, shAFF4#1, or shAFF4#1 restored with CDH1 in the serum-free medium, and migrated **(E)** or invasive **(F)** cells through the 8-μm hole were fixed by 80% cold methanol and stained by crystal violet.

To uncover the AFF4-mediated downstream gene contributes to tumor metastasis, quantitative real-time PCR (qRT-PCR) screened the expression of a panel of multiple EMT-related genes, including *CDH1*, *CAV1*, *ITGB1*, *CDH2*, *MMP9*, *VIM*, *CLDN3*, *FN1*, and *VDR*. As shown in **Figure 5A**, AFF4 depletion significantly induced a decreased expression of *CDH1* while other genes, especially *CAV1*, *ITGB1*, *MMP9*, *VIM*, *FN1*, and *VDR* has a slight change in mRNA level (**Figure 5A**).

*CDH1* as a gene encoded E-cadherin, which is a mark of epithelial cell and indicates loss of the ability to migrate or invade. In HT29 or DLD-1 cells with or without AFF4, we observed that the level of E-cadherin was corelated with the expression of AFF4 (**Figure 5B**). Also, we examined E-cadherin level in HCT116 or LoVo cells before and after AFF4 overexpression, and we found E-cadherin was dramatically upregulated in the cells highly expressing AFF4 (**Figure 5C**). Thus, these data indicated that *CDH1* may be a downstream target gene of AFF4 in CRC cells. To further consolidate this idea, we artificially restored *CDH1* expression in HT29 or DLD-1 cells with AFF4 depletion (**Figure 5D**) and the migration and invasion assay were performed (**Figure 5D**). As shown, the *CDH1* reconstitution in CRC cells with AFF4 depletion led to a deficiency of cell migration and invasion (**Figures 5E, F**); hence, we confirmed that AFF4 served as a tumor suppressor in CRC metastasis *via* upregulating *CDH1*.

## Discussion

Metastasis of malignant tumors does not only determine the degree of malignancy and the patient’s prognosis but is also a key factor leading to the death of the patient and is also the most difficult aspect in the treatment of malignant tumors. For patients with advanced and late-stage diagnosis CRC or multiple organ metastases, the high mortality rate of CRC shows that almost only one in 10 patients can survive for 5 years unfortunately. The distant metastasis of CRC is mainly to the liver. About 50% of patients will have preoperative or postoperative liver metastasis, and about 30% of patients have hidden liver metastasis that cannot be detected by B ultrasound or CT before surgery. However, only a small percentage (10%–20%) are suitable for surgical resection, and even in the early stages of surgical treatment, 80% of colorectal cancer patients will develop liver metastasis within 2 years (20). Although molecular-targeted chemotherapy, new radiotherapy, and interventional therapy have been applied in clinical practice in recent years, the efficacy is still very limited, and liver metastasis is still the main cause of death in CRC patients (21, 22). Therefore, it is urgent to study the molecular mechanism of invasion and liver metastasis of CRC and discover new effective individualized anticancer therapeutic approaches and postreduction indicators, which have important clinical value in improving the survival and prognosis of colorectal cancer patients.

Epithelial-mesenchymal transition (EMT) plays a crucial role in a variety of physiological and pathological processes. It is also a key process in tumor invasion and metastasis (23, 24). Abnormal expression of epithelial E-cadherin encoded by *CDH1* gene is critical in EMT. E-cadherin, a Ca^2 +^-dependent cell adhesion molecule belonging to the Cadherin family, plays a critical role in normal epithelial cell recognition and adhesion, regulation of intercellular connectivity, establishment and maintenance of tissue structure, and integrity of epithelial tissue. Partial or total loss of expression occurs in most human cancers (25, 26). The susceptibility to epithelial tumors is increased or tumor cells are easy to spread and infiltrate due to decreased cell adhesion ability (27–29) although the expression level of E-cadherin did not absolutely match the malignancy of the tumor (30).

The SEC family members have diverse and far-reaching influence on gene expression during normal development and disease pathogenesis. The SEC family of related complexes increases the regulatory diversity and gene control options during transactivation of diverse cellular genes and viruses. AFF4 serves as the scaffold protein of SEC, is mainly studied as a transcription activator that plays a positive role in regulating transcriptional elongation by RNA polymerase II (Pol II), and promotes cell development and differentiation. Otherwise, AFF4 is involved in osteogenic differentiation of human mesenchymal stem cells and odontogenic differentiation of human dental pulp cells and may play a role in regulating pluripotency. Also, AFF4 promoted tumorigenesis and initiation, progression, invasion, and metastasis in leukemia, HIV transcription, and head and neck cancer. This makes SEC or its central component factor AFF4 a broad-range target for cancer therapeutic interventions (8, 12, 31). However, its role in CRC has not been investigated which may offer a more powerful and effective biomarker or therapy target for colon cancer treatment. As a consequence, we give an assumption that AFF4 plays an important role in promoting the expression of E-cadherin encoded by *CDH1* gene and may be a potential strategy for treating CRC.

In the current study, we first tested the gene expression of SEC components, including the scaffold proteins AFF1, AFF2, AFF4, ENL, and AF9 and the elongation factors ELL1 and ELL2. We observed that in CRC tissues, AFF4/1 were downregulated while ENL/AF9/ELL2 were upregulated (**Figure 1A**). The differential expression of these SEC components indicates they would play distinct roles in CRC, or their functions not only existed in SEC but also in other complexes, hence leading diverse influence on gene expression. We further examined the expression of AFF4 in CRC and adjacent tissues because exploring the tumor suppressor could be much more significant for tumor treatment as we have developed some methods to deliver tumor suppressor. Finally, the relationship between AFF4 expression and clinicopathological features was evaluated. We found that AFF4 was significantly downregulated in colorectal cancer tissues and the prognosis of patients with low expression of AFF4 was poor. These data fully demonstrate that AFF4 might be a potential biomarker for tumor progression in colorectal cancer. However, the mechanism underlying the effects of AFF4 on colorectal cancer remains unclear.

Hence, cell models were successfully constructed by AFF4 knockdown and overexpression in our work. The results of *in vitro* experiments then confirmed that AFF4 knockdown or overexpression did not affect the proliferation of colorectal cells, but the absence of AFF4 significantly promoted the migration and invasion of colorectal cells. After overexpression of AFF4, the migration and invasion of HCT116 and LoVo decreased significantly. These results indicated that AFF4 plays an important role in regulating the migration and invasion of colorectal cells. We have identified AFF4 could play a suppressive role in cell migration and invasion *in vitro*. However, given the complexity of the *in vivo* environment, we need further to ascertain its role in hepatic metastasis mouse model. Furthermore, we injected the manipulated cells as AFF4 knockdown CRC cells into the spleen to monitor the metastasis of CRC cells *in vivo*. The spleen-liver metastasis mouse model can simulate the blood flow of liver metastatic cells, especially the progression of cell migration and invasion from primary colorectal cancer. The final results confirmed that AFF4 deficiency enhanced the capacity of colorectal cancer cells to metastasize *in vivo*. However, the downstream target of AFF4 is still unknown.

We then detected the expression of CDH1 protein in HT29 or DLD-1 cells with AFF4 depletion and HCT116 or LoVo cells with AFF4 overexpression ulteriorly; the results showed that once AFF4 was depleted, CDH1 protein was then downregulated. However, CDH1 was significantly upregulated after overexpression of AFF4, suggesting that CDH1 may be the downstream target of AFF4 in CRC cells. To further consolidate this view, we first artificially restored the expression of CDH1 in HT29 or DLD-1 cells with AFF4 depletion and then identified that CDH1 could inhibit cell migration and invasion through constructed cells. Therefore, we enhanced the anticancer effect of AFF4 in CRC.

In conclusion, the present study displayed that AFF4 is downregulated in CRC tissues, which is inversely predicted with poor prognosis, revealing the key role of AFF4 in colorectal cancer. Dysregulation of the transcriptional elongation stage is implicated in solid tumor, suggesting that understanding the mechanism of transcription elongation is therapeutically relevant for cancer therapy. Current data suggest that the role of AFF4 is associated with clinicopathological features of CRC and involved the *in vitro* and *in vivo* invasion and metastasis of CRC cells. Overall, our results call attention to the unrecognized potential role of AFF4 in CRC and contribute to the identification of AFF4 as a prognostic biomarker, adding an attractive and challenging therapeutic target to the search for new therapeutic prospects for CRC patients.

Still, there are several limitations in this study. Firstly, how AFF4 plays as a transcription activator to inhibit migration and invasion of CRC, the upstream molecular mechanisms of AFF4 in CRC remain largely unknown and need further study. Secondly, AFF4 promotes malignancy`s initiation and progression in the reported diseases such as leukemia, head and neck cancer, and bladder tumor. In this study, AFF4 is downregulated in the CRC tissues and reversely predicts poor prognosis and overexpression of AFF4 decreased the ability of CRC metastasis. How to reasonably improve AFF4 expression in CRC cells to an acceptable level to inhibit CRC metastasis without heavily disturbing other physiological and pathological homeostasis remains a major challenge. Thirdly, the initiation and progression of CRC consist of several stages. Repeated sampling and longitudinal monitoring of the development of CRC can ameliorate our understanding of the role of AFF4 in the physiological and pathological tissue transformation and exploring the specific stage of AFF4 in inhibiting colorectal cancer is crucial for the development of targeted therapies for CRC and other cancers.

## Data Availability Statement

The datasets presented in this study can be found in online repositories. The names of the repository/repositories and accession number(s) can be found in the article/**Supplementary Material**.

## Ethics Statement

The studies involving human participants were reviewed and approved by the Ethics Committee of Ruijin Hospital, Shanghai Jiao Tong University School of Medicine, Shanghai, China. The patients/participants provided their written informed consent to participate in this study. The animal study was reviewed and approved by the Ethics Committee of Ruijin Hospital, Shanghai Jiao Tong University School of Medicine, Shanghai, China.

## Author Contributions

Conception and design: XPJ and HP. Development of methodology: YF and XQJ. Acquisition of data (provided animals, acquired and managed patients, provided facilities, etc.): YF, XG, and XQJ. Analysis and interpretation of data (e.g., statistical analysis, biostatistics, computational analysis): HC, YZC, SZ, and YJ. Writing, review and/or revision of the manuscript: YF, XQJ, YQC, and YZ. Administrative, technical, or material support (i.e., reporting or organizing data, constructing databases): XQJ, YJ, YQC, and YZ. Study supervision: XPJ, HP, and XQJ. All authors listed have made a substantial, direct, and intellectual contribution to the work and approved it for publication.

## Funding

This study was supported by National Natural Science Foundation of China [81902374(XQJ)], [81870011(WGC)]; the Youth Development Program of Shanghai Tenth People’s Hospital [04.03.19.140(YF)].

## Conflict of Interest

The authors declare that the research was conducted in the absence of any commercial or financial relationships that could be construed as a potential conflict of interest.

## Publisher’s Note

All claims expressed in this article are solely those of the authors and do not necessarily represent those of their affiliated organizations, or those of the publisher, the editors and the reviewers. Any product that may be evaluated in this article, or claim that may be made by its manufacturer, is not guaranteed or endorsed by the publisher.

## Abbreviations

AFF4, AF4/FMR2 family member 4; CRC, colorectal cancer; SEC, super elongation complex; EMT, epithelial-mesenchymal transition.
