# Supplementary material for: AFF4 Predicts the Prognosis of Colorectal Cancer Patients and Suppresses Colorectal Cancer Metastasis via Promoting CDH1 Expression
Source: Front Oncol. 2022 Feb 9;12:797392. doi: 10.3389/fonc.2022.797392 (PMC8865618; doi:10.3389/fonc.2022.797392)
Supplement: Supplementary file 1 [file DataSheet_1.docx]

**
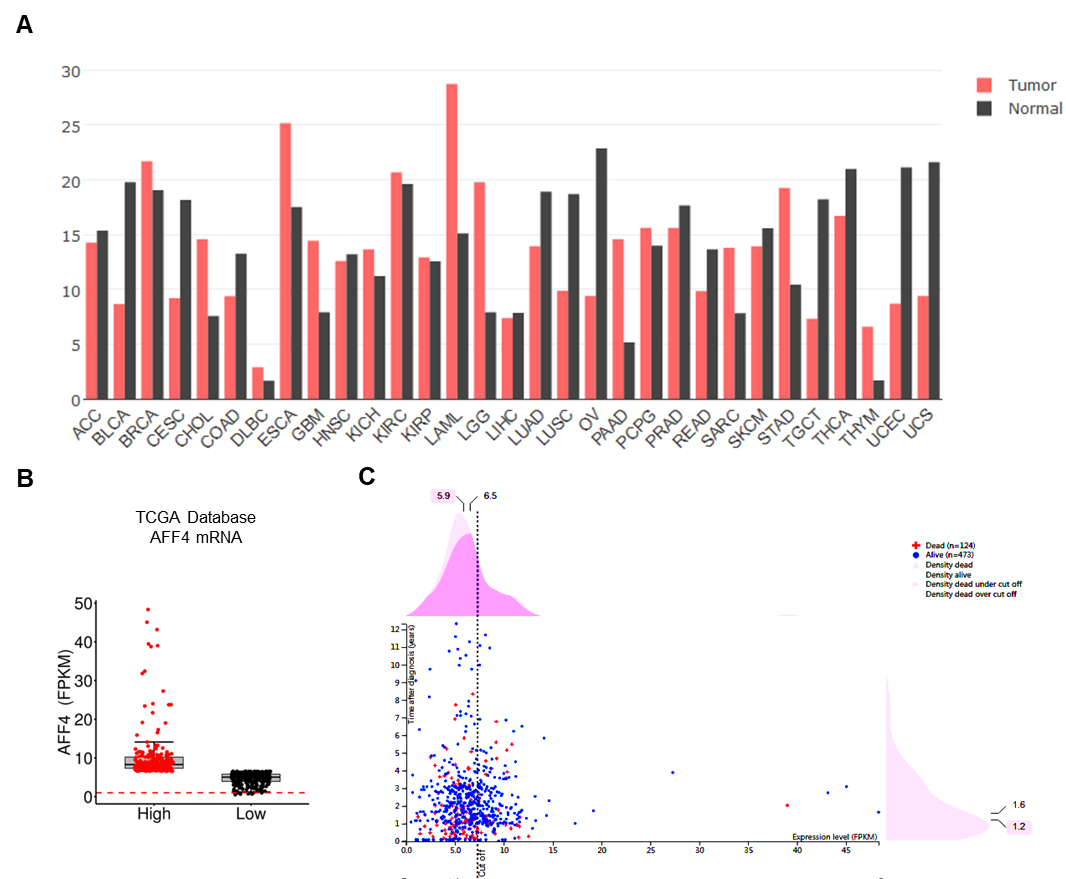
Supplementary Figure and Legends**

**Supplemental Figure 1.**

A. The gene expression of *AFF4* in 31 tumor tissues and their related adjacent normal tissues was extracted from GEPIA database. Red marks tumor sample while Black marks normal tissue sample.

B. The FPKM value of AFF4 was presented as a median cutoff. All of the information about CRC patients was extracted from TCGA database.

C. The FPKM value of AFF4 was presented as a best cutoff. All of the information about CRC patients was extracted from the human protein atlas.


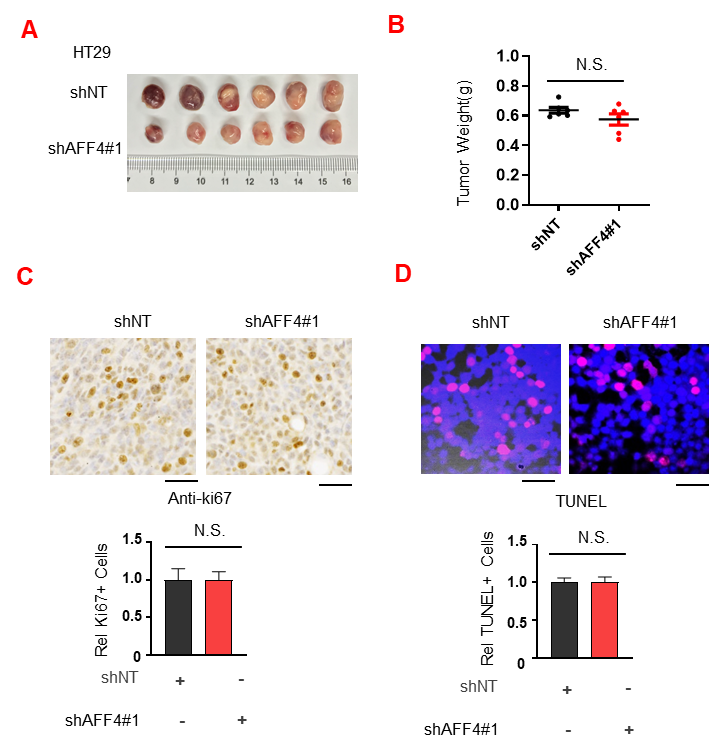


**Supplemental Figure 2.**

A-D. Xenograft study. The stable cell lines derived from HT29 expressing shNT or shAFF431 were transplanted into the left groin of nude mice (n=5, each group). Solid tumors were dissected and imaged at the end of the experiment (A) and tumor weight was measured (B). (F) Ki67 (C) or TUNEL staining (D) was performed to examine proliferating or apoptotic cells in solid tumors. Scale bar, 100 μm. C and D, two-tailed Student’s t-test.

**Supplementary materials and methods**

**Cell Culture**

HT29, DLD-1, HCT116, and LoVo cells were purchased from American Type Culture Collection (ATCC, Manassas, VA, USA). All cells were cultured in DMEM basic supplemented with 10% fetal calf serum (Gibco, Australia origin). Total RNA was extracted and isolated from tissue samples and cell lines using Trizol reagent (Invitrogen, Carlsbad, CA, USA). Reverse transcription of RNA was performed using the reverse transcription kit (Promega, Beijing, CHN).

**Western Blot Analysis**

The cells were lysed with RIPA buffer (Cell Signaling Technology, Danvers, MA), and the protein concentration was measured by using the BCA assay (Thermo Fisher Scientific, Waltham, MA). Equal amounts of protein were subjected to 10% SDS-PAGE for electrophoresis and transferred to a polyvinylidene difluoride membrane (Millipore, Billerica, MA). The membrane was blocked with 5% fat-free milk at room temperature and then incubated with specific anti-AFF4 primary antibody (Novus, NBP1-81504, dilution 1:1000) and anti-GAPDH (Thermo Fisher, Waltham, MA, MA5-15738, dilution 1:1000) antibodies overnight at 4°C. Antibodies against AKT1 (#4685, 1:1000), pAKT-S473 (#4060, 1:1000), YAP (#14074, 1:1000), pYAP-S127 (#13008, 1:1000), ERK1/2 (#5013, 1:1000) and pERK1/2-T202/204 (#4370, 1:1000) were purchased from Cell Signaling Technology. Immunoreactive bands were visualized with enhanced chemiluminescence (Thermo Fisher Scientific, Waltham, MA) by using the LAS 4000 Imaging system (Fujifilm, Tokyo, Japan).

**Immunohistochemistry (IHC) analysis**

Tissue sections from paraffin-embedded human CRC tissues were stained with antibodies as indicated. We quantitatively scored tissue sections according to the percentage of positive cells and staining intensity. We rated the intensity of staining on a scale of 0–3: 0, negative; 1, weak; 2, moderate; and 3, strong. We assigned the following proportion scores: X indicates the percentage of tumor cells that were stained (0 ≤ [X1 + X2 + X3] ≤ 100), where X3 indicates strong staining, X2 moderate staining and X1 weak staining. The score (H-score) was obtained using the following formula: 3 × X1 + 2 × X2 + 1 × X3, giving a range from 0 to 300. Scores were compared to overall survival, defined as the time from the date of diagnosis to death or last known date of follow-up. Use of human CRC specimens was approved by the IRB at Shanghai Ruijin Hospital and complied with all relevant ethical regulations. Informed consent was obtained from all patients. Indicated antibodies against AFF4, Ki67 and TUNEL were used to perform IHC.

**Supplementary Table 1**

**Primers for constructs：**

UNI-CMV-AFF4-F

GTGGAATTCGCTAGCGGATCC atgaaccgtgaagaccggaat

UNI-CMV-AFF4-R

ATGTCGACCTCGAGTGCGGCCGC tcaagatatcaacttggcatc

**Sequences of Primers for qRT-PCR**

Homo-AFF4-F 5'- TCTCAGTCTCAGAAACGGTCC

Homo-AFF4-R 5'- GGCTACTGCTCCCACTATTGTT

Homo-CDH1-F 5’-ATTCTGATTCTGCTGCTCTTG

Homo-CDH1-R 5’-AGTAGTCATAGTCCTGGTCTT

Homo-CDH2-F 5’-TCAGGCGTCTGTAGAGGCTT

Homo-CDH2-R 5’-ATGCACATCCTTCGATAAGACTG

Homo-CAV1-F 5’-CATGCCTGTCATACCACAAC

Homo-CAV1-R 5’-GGTGTCAGATGGAGGAGGG

Homo-ITGB1-F 5’- CCTACTTCTGCACGATGTGATG

Homo-ITGB1-R 5’- CCTTTGCTACGGTTGGTTACATT

Homo-MMP9-F 5’-T GTACCGCTATGGTTACACTCG

Homo-MMP9-R 5’- GGCAGGGACAGTTGCTTCT

Homo-VIM-F 5’-ATTGCCACCTACAGGAAGCT

Homo-VIM-R 5’-GCAGAAAGGCACTTGAAAGC

Homo-CLDN3-F 5’- AACACCATTATCCGGGACTTCT

Homo-CLDN3-R 5’- GCGGAGTAGACGACCTTGG

Homo-FN1-F 5’- AAGATAACCGTGTGATGCAGTT

Homo-FN1-R 5’- GGGGAGCAGGTAATGACGTATTT

Homo-VDR-F 5’- GTGGACATCGGCATGATGAAG

Homo-VDR-R 5’- GGTCGTAGGTCTTATGGTGGG

Homo-ACTB-F 5’-TGGACTCTGTTCGCTCAGGT

Homo-ACTB-R 5’TGCCTCCTTCCGTACCACAT
